# Stress Induced Mechano-electrical Writing-Reading of Polymer Film Powered by Contact Electrification Mechanism

**DOI:** 10.1038/srep19514

**Published:** 2016-01-20

**Authors:** Sumita Goswami, Suman Nandy, Tomás R. Calmeiro, Rui Igreja, Rodrigo Martins, Elvira Fortunato

**Affiliations:** 1 i3N/CENIMAT, Department of Materials Science, Faculty of Science and Technology, Universidade NOVA de Lisboa and CEMOP/UNINOVA, Campus de Caparica, 2829-516 Caparica, Portugal

## Abstract

Mechano-electrical writing and reading in polyaniline (PANI) thin film are demonstrated via metal-polymer contact electrification mechanism (CEM). An innovative conception for a non-destructive self-powered writable-readable data sheet is presented which can pave the way towards new type of stress induced current harvesting devices. A localized forced deformation of the interface has been enacted by pressing the atomic force microscopic probe against the polymer surface, allowing charge transfer between materials interfaces. The process yields a well-defined charge pattern by transmuting mechanical stress in to readable information. The average of output current increment has been influenced from 0.5 nA to 15 nA for the applied force of 2 nN to 14 nN instead of electrical bias. These results underscore the importance of stress-induced current harvesting mechanism and could be scaled up for charge patterning of polymer surface to writable-readable data sheet. Time evolutional current distribution (TECD) study of the stress-induced patterned PANI surface shows the response of readability of the recorded data with time.

Charge transfer phenomenon at the metal-polymer interface by contact electrification is among the most exciting physical phenomena, both as a source of information on materials physics and also due to its huge scope in nanoelectronics and device applications. Contact electrification, which was discovered around 600 BC, is still a subject of intense research on the basis of fundamental materials research and also for an alternative way of harvesting energy in newly emerging fields of mechano-electronics^1^,^2^,^3^,^4^,^5^. Contact electrification can be considered as a phenomenon of charge transfer and back transfer (which may be a single type of mobile ions, electrons or a mixture of ions and electrons) between the surface of two different materials through the Fermi level energy barrier when they are brought into contact and separated (with or without intentional rubbing or frictional contact)^6^,^7^. Depending on the height of the energy levels charge carriers are accepted or donated from the metal surface. However, the effect has been regarded as one of the factors for undesirable power dissipation in electronic circuits, but recent ideologists in materials science are studying this outcome in contextual applications like painting^8^,^9^, particle separation^10^,^11^,^12^, polymerizations^13^,^14^,^15^, sensors^16^ mechano-electrical data storage and energy harvesting^17^,^18^,^19^ etc. Contact electrification can be classified according to the type of the solid surfaces involved: metal-metal, metal-insulator and metal-semiconductor system. Though in comparison to the metal-semiconductor system, the first two systems have been extensively used in contact electrification mechanism or in terms of triboelectrification for the last two decades. Here, we propose a conceptually new design to write and read of memory bits on polymer surface using mechano-electrical response instead of applying any electrical bias. Through converting mechanical stress into readable information powered by contact electrification mechanism (CEM), such polymer surface could be scaled up as a nanoscopic writable-readable data sheet.

Towards exploring these mechano-electrical energy harvesters, especially for polymer based systems, one major issue is the selection of material. Polymer material comprises significant advantages over the conventional inorganic material based electronics due to its attractive features including miniaturized dimension and feasible improvisations in physical properties through molecular design and chemical synthesis. In particular, among all the polymers, much attention has been paid to conjugated polymers (also known as organic semiconductors or conducting polymers) for organic based electronic devices (OED) due to their interesting opto-electronic configuration, which can be varied from insulator to metal like behaviour^20^,^21^. A central issue in the physics of these π-conjugated polymers (and the corresponding oligomers) is the strong coupling between electronic, geometric and chemical structures, *i.e.*, the bonding pattern of atoms in the molecular system. It is increasingly clear that the electronic properties of conjugated polymers depend sensitively on the physical conformation of the polymer chains and the way the chains pack together in films. Among these conjugated polymers, polyaniline (PANI) is one of the most investigated because of its facile synthesis, excellent electronic properties and high environmental stability^22^,^23^. A distinctive feature of PANI is the reversibly tunable redox characteristics that allow the control of electrical conductivity over a wide range by protonation and charge-transfer doping^24^. Eventually, the polaronic structure in protonated emeraldine form of PANI makes it interesting to study as a conducting polymeric material. The presented work has evidenced unique contact electrification of this polymeric film at its metal-polymer interface. The signification of this article is to offer a deep insight into this phenomenon using polaron based charge transfer mechanism of PANI and to further evolve this idea into the formation of polymer based mechano-electrical nanoscopic writable-readable data sheet.

## Results

**Figure 1:**
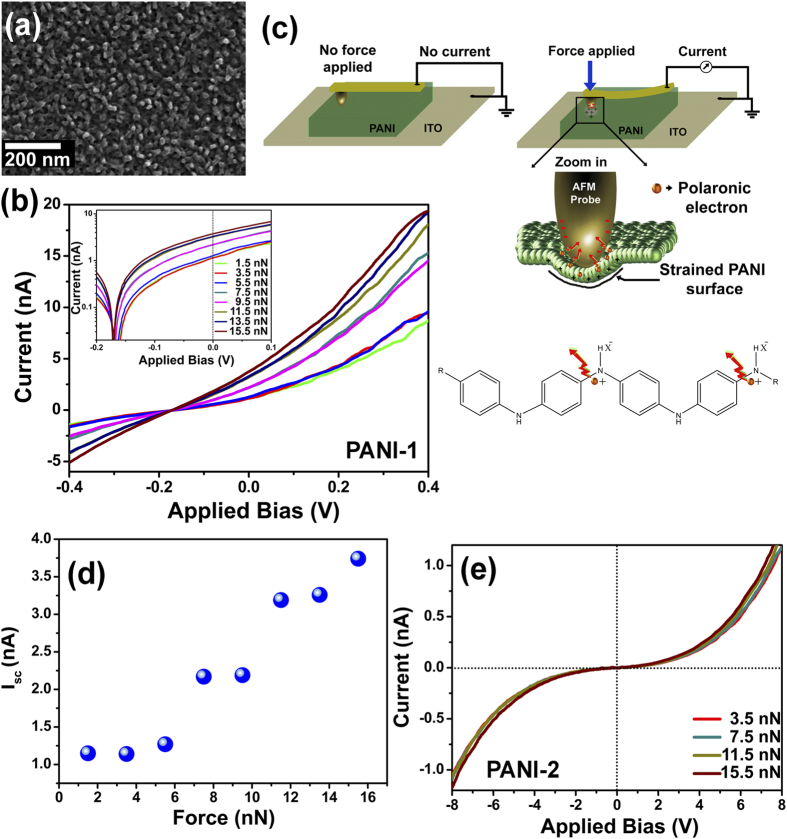
(**a**) FESEM image of the polyaniline thin film deposited on ITO coated glass substrate. (**b**) The asymmetric modulation of current transport dynamics at the metal-polymer (PANI-1)-metal interface under different applied contact force showing stress-induced characteristics. **Inset:** Corresponding semi-logarithmic plot clearly demonstrates a current generation at 0 V applied field (*I*_*SC*_). (**c**) Schematic illustration of the experiments based on CEM demonstrating a charge generation at the presence of applied force. Localized electron transfer has been initiated at the metal-polymer interface under typical stress. Contacting surface stretching has been illustrated through zoom. Electrons that reside on the amine group of ES-PANI (protonated form of polyaniline), may gain enough energy to surmount the localized interaction and initiate to transform to the nearest lower energy band *i.e.* to metal body. (**d**) Dependence of the short circuit current (*I*_*SC*_) on the contacting force applied by AFM probe. Larger contacting force produces higher *I*_*SC*._ (**e**) *I-V* characteristics of more oxidized form of EB (namely, PANI-2), exhibits no short circuit current.

Polyaniline thin films were deposited on ITO coated glass substrate by a chemical oxidative polymerization method. The localized electron transport behaviour has been investigated based on contact electrification mechanism at metal-polymer-metal interfaces, where ITO acted as a bottom electrode and *Pt-Ir* coated AFM probe was used as localized top electrode. CEM has been explored by *in-situ* atomic force microscope technique in contact mode by using a metal coated probe with different applied forces. Detailed fabrication method of PANI thin films on ITO electrode is given in Experimental Section. Figure 1(a) shows a field emission scanning electron microscopy (FESEM) image of the thin film surface, revealing that PANI forms a tiny network cross-linked with adjacent nanostructures. A close look at the nanostructure growth shows a compact PANI film. Confirmation of PANI has been described extensively by FTIR analysis, given in Fig. S1(a) in supplementary information. Also all the deposited forms of polyaniline films were amorphous in nature. The corresponding XRD of different form of PANI films have been displayed in Fig. S1(b) in supplementary information. In order to develop mechano-electrical response based on metal-polymer interface CEM, we have first studied the *I-V* characteristics of the PANI thin film at a particular point by AFM probe using different probe-surface contacting forces. The AFM probe was calibrated before each measurement with all the factors that affect the data acquisition kept fixed. Figure 1(b) exhibits the *I-V* characteristics for an applied voltage range from +0.4 to −0.4 V with increasing probe-surface contacting force. The corresponding semi-logarithmic plot of which in the inset of Fig. 1(b) clearly demonstrates a current generation at 0 V applied field (can be claimed as short circuit current, *I*_*SC*_). According to the conventional theory of CEM, when the top electrode lands on the polymer surface, a localized stress is developed on the contact surface area, generating different charge distributions within the polymer-metal interface. The stretched surface exhibited localized charges (shown in the scheme in Fig. 1(c)) compared with the un-stretched surface, initiating charge carriers (electrons or ions) to transfer from metal to polymer or vice-versa, due to the difference in Fermi energy level of two materials^25^. Contact charging has been considered as a non-equilibrium two-step mechanism, consisting of instantaneous bond-breaking process and bond-forming process^26^. Interestingly, our study actualizes the fact of charge transfer and back transfer mechanism at the metal-polymer interface at the time of contact. However, the dynamics of charge transfer mechanism for a semiconducting polymer like polyaniline is a subject of intense research. An explicit investigation on the current characteristics shows that, other than the emeraldine salt (ES) form, there was no short circuit current (*I*_*SC*_) for other different forms of polyaniline, i.e., fully reduced (leucoemeraldine base; LB) and fully oxidized (pernigraniline base; PNB) systems based on CEM. This contextual outcome imparts that contact charging is not solely due to the transfer of charge carriers (i.e. electrons, ions or both) between the Fermi levels of two surfaces, but entails spatially certain electronic structure of polyaniline in conducting form.

**Figure 2:**
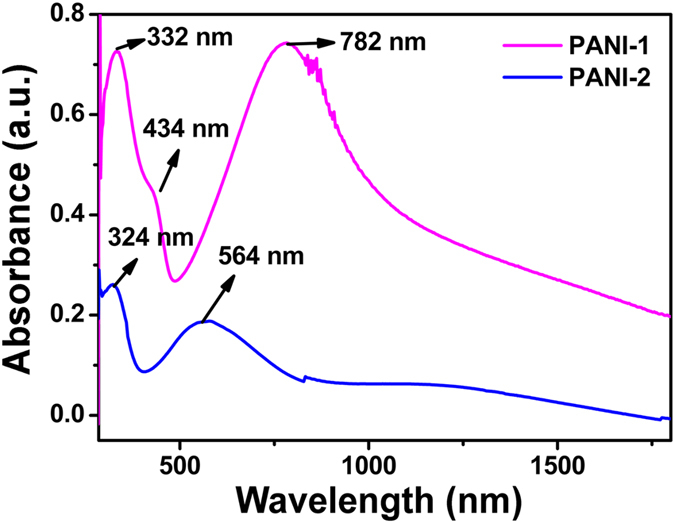
UV-Vis spectra of the protonated form of PANI (PANI-1) and the more oxidised form of PANI (PANI-2). Spectrum of PANI-1 shows two bands with *λ*_max_ = 332 and 782 nm, which are assigned to the *π*-π^*^ transition of the benzenoid rings and the excitation absorption of the quinoid rings, respectively. A small shoulder detected at *λ*_max_ = 434 nm is attributed to the formation of polarons. Red shift in PANI-2 indicates more oxidised form of polyaniline.

The LB form of polyaniline is formed by benzene rings which are connected by a saturated amine linkage, inhibiting conjugation, therefore acting as insulator. Previous experimental studies found that the maximum in π-π^*^ optical absorption spectrum in the LB form is 3.8 eV, which is far wide^27^. As the intrinsic redox states associated with the nitrogen atoms are unique to PANI and act as a prevailing role on the doping behaviour which further attributing to the charge transfer properties^28^. Thus the current generation due to contact electrification has been shown for protonated emeraldine base PANI *i.e.* ES-PANI. As synthesized PANI consists of alternating reduced (amine group; 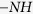) and oxidized (imine group; −*N*=) repeat units, known as the emeraldine base (EB) form. However, after the treatment with sufficient acid dopant (HX), EB-PANI becomes protonated and transforms into the conductive ES form. The protonation of this form create polarons, the charge carriers able to travel along the macromolecule backbone or even to move on neighboring macromolecules through variable range hopping (VRH) mechanism^29^. Thus, protonation of PANI led to a significant increase in charge transfer mechanism. Therefore, electrons that reside on the amine group of ES-PANI, may gain enough energy to surmount the localized interaction and initiate to transfer to the nearest lower energy band. A schematic of the mechanism has been illustrated in Fig. 1(c). The number of electron transfer will increase with the increasing localized stress on the polymer surface induced by the metal probe. Figure 1(d) shows the dependence of short circuit current (*I*_*SC*_) on different applied forces by AFM probe on the PANI surface. The *I*_*SC*_ exhibits an increasing order of value with the raise in applied force, *i.e*., with the increasing amount of stress developed at the PANI surface for protonated EB or ES form (namely, PANI-1). The experimental data shows that the PANI surface exhibited an 11.4% increment in *I*_*SC*_ for an applied force ranging from 1.5 nN to 5.5 nN. Further enhancement in applied force yields a sharp increment in *I*_*SC*_. Result shows that the electric output is strongly related to the contact force, yielding higher output with larger force. But the same behaviour was not observed for more oxidized form of EB (namely, PANI-2). Figure 1(e) depicts a characteristic *I-V* of the PANI-2 film for different probe bias, yielding no current generation at 0 V. Above results impact on the polaronic structure of PANI, which is almost unavailable for over oxidized sate of PANI (due to the formation of more bipolaronic structures in polymer chains). Figure 2 shows the UV-Vis spectroscopy of these two samples with different oxidation states, PANI-1 and PANI-2. A small satellite has been detected at 434 nm and the broad peak near 782 nm is attributed to the formation of cation radicals (polarons), indicating the doped state of the sample. The broadening at 782 nm arises due to electronic delocalization which may lead to the charge transfer. The red shift of the broad peak with absence of satellite peak indicates the absence of delocalized electronic structures in the second sample, which can be signified to the increase in oxidation level of PANI^30^. Also the FTIR of two samples agreed with the UV-Vis spectra. This leads to our assumption that the contact electrification was initiated for the PANI thin film due to the delocalized electronic structure. Moreover, this form of PANI leads to some defects which may also generate the electron transfer. As both the PANI-1 and PANI-2 films are amorphous in nature, hence the crystallinity does not play any significant role to affect in electron transfer mechanism.

**Figure 3:**
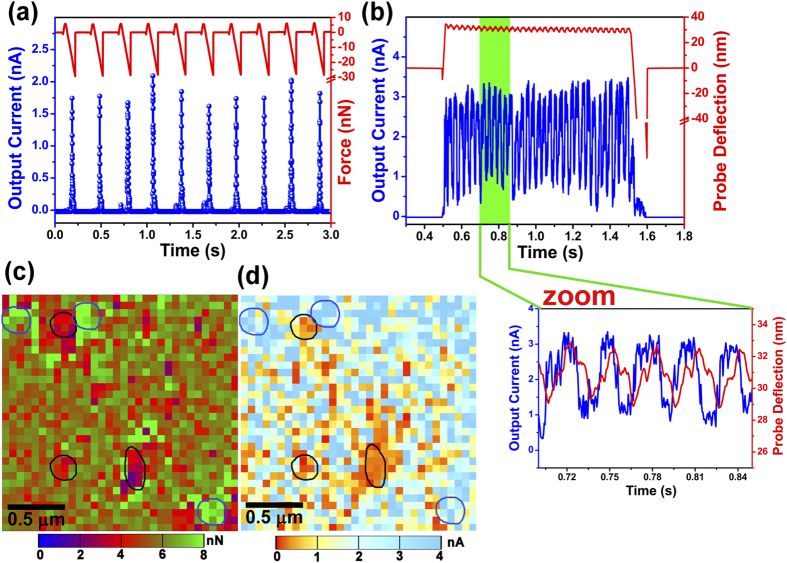
(**a**) Exhibits successive performance of current output from the PANI surface for each contact with AFM probe under an applied force of 5.5 nN. Current response time is approximately 0.05 s. (**b**) Output current measured with respect to time under an constant force. Probe deflection during the contact with polymer surface shows a wavelet nature with 2 nm in amplitute. Enlargement of the current pulse (section in green) illustrates similar wavelet envelope as the probe deflection, which can be considered as molecular vibration during electron tunneling at metal-polymer interface. (**c**) Adhesion map (32 × 32 matrix with a step pixel size of 40 nm) of the PANI-1 surface. Domain in blue and black show some typical regions, measured with high and low force of adhesion. (**d**) Simultaneous measured map of electrical output. Corresponding domain with adhesion map shows higher to lower regions of output current, attributed to strain dependent electrical output.

The electrical outputs with respect to time under successive stresses by AFM probe has been shown in Fig. 3(a). The corresponding current peaks (blue in figure) can be observed for the particular stress period (~0.1 s), which has been applied to PANI surface by AFM probe. When the stress is released current sharply reduces to zero. We have controlled the contact electrification characteristic system for 10 consecutive repetitive landing of AFM probe on the polymer surface for different time spans. With each landing of AFM probe, the surface of contact is getting stretched and released accordingly and current outputs are recorded respectively. The probe sample contact area is typically less than 20 nm in radius, so switching can be highly localized, allowing charge transfer at the native domain to the metallic probe where no voltage is applied during mechanical switching. The typical electrical outputs were in average approximately 1.8 nA. Each and every localized hit by the AFM probe at the contact area generates current by initiating the electron transfer. This phenomenon of current generation through the application of stress on the polymer surface is part of mechano-electrical effect. The electrical outputs were also tested for the short period of the stress-hold-release states. During the stressed process, the average current output was about 3 nA for 5.5 nN applied force. Interestingly, observed current outputs in Fig. 3(b) are intermittent in nature. A close look at the deflection of AFM probe (green slice in Fig. 3(b)) indicates a sinusoidal drifting. Fluctuations of about 2 nm of wavelet amplitude in probe deflection during the contact between AFM probe and polymer surface has been observed. A probable explanation can be exerted to both, molecular vibration during electron tunneling at metal-polymer interface and the vibrations in local structure due to inter-molecular electron transition (HOMO to LUMO) within the polymer. These fluctuations in turn cause an effect on mechano-electrical process at the interface layer by nanoscopic resolution with discrete output of generating current spectra.

**Figure 4:**
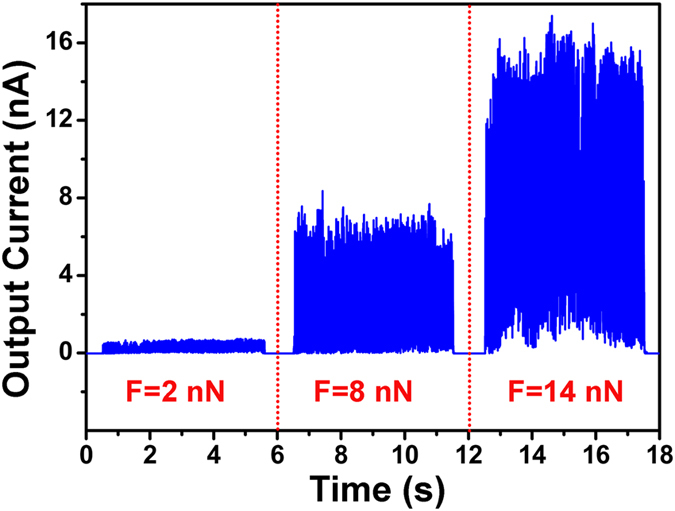
The measured electrical response for typical three different contacting forces of 2, 8 and 14 nN. Each applying force was conferring for 5 s. Electrical response was increased approximately from 0.5 to 15 nA with enhancement of applying force.

To establish the presence of stress-induced response in surface conductance, the PANI surface was simultaneously mapped for localized adhesion and yield of current. The measurement was carried out without applying any voltage to the probe. The adhesion map of material surface is obtained by bringing the AFM probe in contact with the sample and then retracting it. The pull-off (adhesion) force is measured as the minimum tension required to detach the tip from the sample surface. The nature of the adhesion can be associated with different types of attraction forces mainly van der Waals and electrostatic forces, taking into consideration the measured value of the pull-off forces^31^,^32^. In addition, as the applied probe force is constant throughout the mapping, AFM-determined adhesion behaviour of these polymeric materials may be due to the increased area of interaction between the tip and the probed surface, resulting from localized surface deformation of the polymer surface. First approach to the localized map of PANI surface sites had the objective of recording arrays of force curves in the *x,y* plane using a metal coated probe and measuring the corresponding current outputs. Typically, a 32 × 32 matrix was recorded throughout areas of 2 × 2 *μ*m^2^ in size. The measurements were conducted using a step pixel size of 40 nm. At each point, the current response was recorded as a function of the probe stress. Figure 3(c,d) depict the map of force experienced by the PANI surface with the corresponding stress-induced harvesting of current respectively. The stronger adhesion regions are displayed by the greenish colour where some of the areas are indicated by circles in blue (Fig. 3(c)), that represent regions with higher stress. When the probe is close to the elevated face of the PANI surface, the contact area interacting with the tip increases which develops a stretching in the localized domain resulting in an enhancement in the measured force of adhesion. The presence of such features highlights the complementary nature of output current measurements. The mapping of corresponding regions also illustrates the high current regions in Fig. 3(d) (some of the selected similar areas are indicated by blue circles). By similar reasoning, closely associated areas of low adhesion (reddish area in Fig. 3(c)) may be attributed to the comparatively lesser stretching surface, that witnessed low current (in Fig. 3(d)). It appears that the overall current output intensities of the surface of the PANI films are associated with the strength of surface adhesion, indicating stress-induced current harvesting nature of PANI thin films. These outcomes are in well agreement with the *I-V* measurements. Figure 4 shows a systematic investigation on current outputs for incessant contact mode (~5 s) throughout different values of applied force. Different applied forces show different output currents, which is an indication of the tendency of current generated by the PANI surfaces to increase with a similar increase in the contact force triggered by the probe. The average of output current has been increased from 0.5 nA to 15 nA when the applied force was increased 2 nN to 14 nN. Additional data has been displayed in Fig. S2 of supplementary information.

### Application towards mechano-electrical writing-reading

**Figure 5:**
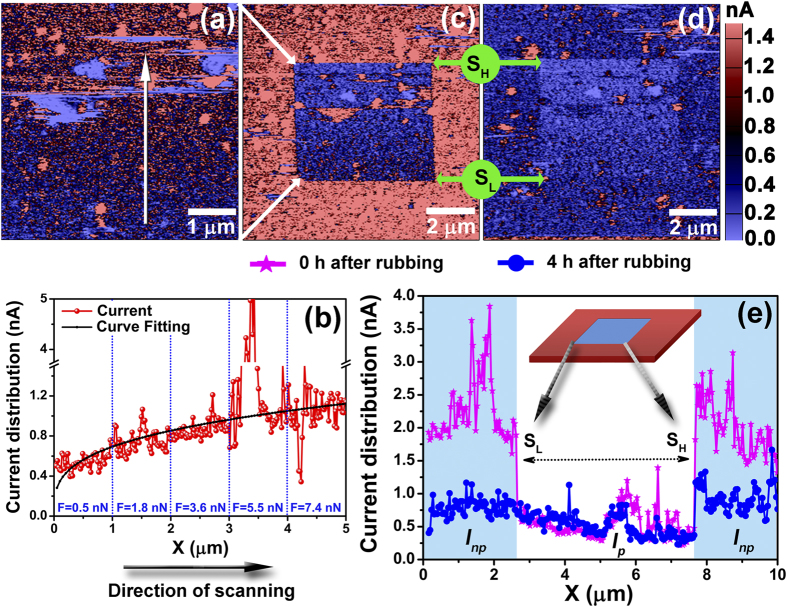
(**a**) Exhibits an accumulation of currents from the PANI-1 surface while ploughing by AFM probe in contact mode. White arrow in the figure indicates the scanning direction with five steps of applied contact force, increasing from 0.5 nN to 7.4 nN. (**b**) Corresponding current distribution profile, exhibits growing nature in current harvesting with increment of applying force. (**c,d**) Exhibit discriminating current distribution throughout the scanned area which sprouts the ploughed area (5 × 5 μm^2^) as readable stuff. The images were scanned after 0 and 4 h respectively. Diversity in current distribution demonstrates that discrete data can be recorded under different applied stresses. (**e**) Current distribution profile for corresponding images. Pink curve is for (**c**) and blue for (**d**). Derived current distribution reflects that the ploughed area yields scanty electrical output (~0.5 nA) where the neighbour yields much excessive (~1.8 nA). Electrical output ratio 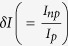 varies at point S_H_ and S_L_. Where “S_H_” and “S_L_” are the positions for higher and lower contacting force induced area. “*I*_*p*_” and “*I*_*np*_” are the output current of ploughed (**a**) and non-ploughed (**c,d**) area.

The second part of our research aims at exploring this stress induced mechano-electrical current harvesting nature into direct writing-reading of polymer applications. In our indicative experiment, the first approach was to plough a 5 × 5 *μ*m^2^ area of a PANI thin film with an AFM probe in contact mode with different contacting forces. The experiment was performed while the back electrode (ITO) was grounded and the AFM probe was acting as the top electrode. During the scanning by AFM probe with particular contact force on the surface area of PANI, contact electrification mechanism has been developed, yielding of current which was recorded simultaneously through the metal-polymer-metal interface structure. The harvesting of current during the ploughing has been illustrated in Fig. 5(a) (white arrow in the figure indicates the scanning direction). The contact force applied on the polymer surface during the ploughing was gradually increased in five steps from 0.5 nN to 7.4 nN along the scanning direction (*i.e.*, the scanning was started with lower contact force and increased gradually after each 1 *μ*m step in the vertical direction). Profile of the corresponding image in Fig. 5(b) exhibits an increment of the harvesting current with an increase of contact force. In pursuit of this stress-induced CEM towards the writing ability on this polymer surface, we have scanned the PANI thin film with a larger area (10 × 10 *μ*m^2^) keeping the ploughed area within. Figure 5(c) shows a clear change in current distribution throughout the scanned area which sprouts the ploughed area as readable stuff. This scanning image was taken just after Fig. 5(a). The image reflects that the ploughed area yields scanty current harvesting (~0.5 nA) where the neighbour yields much more current (~1.8 nA). Both the ploughed and non-ploughed areas are showing a distinguished current distribution that can be employed as a data recorder. The phenomenon can be explained by CEM. When the metal coated AFM probe was brought into contact with the PANI surface by specific contact force, the electrons bound on the amine (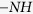), getting sufficient energy to transfer to the metal through polymer-metal interface layer, thus harvesting current without any applied voltage. The equilibrium in Fermi level energy at the interface and thermal agitation due to localized stress may also attribute to the notion. Now, if an electron is removed (transfer) from the PANI surface at the localized interface position (metal-polymer), the scarcity of charges (electron) will create an electric field in the space between the polymer surface and the metal electrode, which may have impeded further carrier tunneling at the metal-polymer interface. Consequently, the factor of current harvesting at the ploughed area will be diminishing in nature. With time the scarcity of these charges at the ploughed area will be filling up by several ways: (a) the backflow resulting from the redistribution of charges due to the conjunction carrier transportation within conducting polymer, (b) back-charge transfer from metal to polymer layer or (c) intra-charge transfer within the polymer from HOMO to LUMO. Distribution of electrical outputs in the ploughed area is also affected by the strain. From the profile of the same cross section of Fig. 5(b) as shown in Fig. 5(e) (curve in pink colour), a well-like distribution of electrical outputs was observed. The explicit steps of electrical outputs have been displayed at extent of ploughed (*I*_*p*_) and non-ploughed (*I*_*np*_) area. We can term this electrical output ratio as 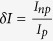 applied over the steps. Stress from lower to higher (indicated by S_L_ and S_H_ respectively in Fig. 5(e)), *δI* of 3.3 and 6.8 were experimentally measured. The electrical output distribution over the same area has been recorded after 4 h, shown in Fig. 5(d). Corresponding profile analysis shows *δI* of 1.0 and 2.9 respectively from lower to higher applied strain (blue in Fig. 5(e)). The time dependent comparison of two profiles indicates that the current harvesting from the ploughed area is both stress and time dependent. Taking this into account, here we have proposed a conceptually new design of stress-induced electrically controllable polyaniline based self-powered data sheet, where writable and readable data has been recorded by controlling the current harvesting from the polymer surface through a stress dependent CEM.

**Figure 6:**
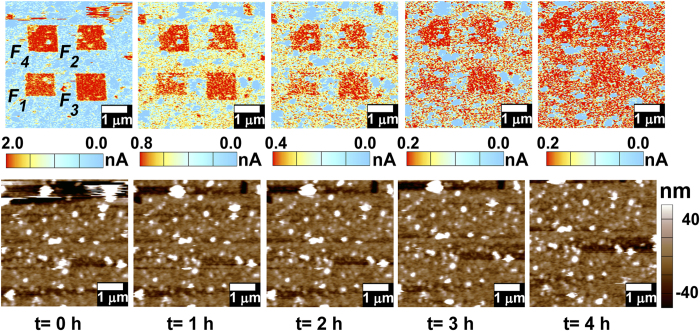
Top: Displays series of time evolutional current distribution (TECD) of four separate domains (1 × 1 μm^2^), ploughed under different contacting force (F1 = 1.5 nN, F2 = 3 nN, F3 = 4.5 nN and F4 = 6 nN). TECD has been recorded for time interval from t = 0 to 4 h with interval of 1 h. A clear diversity of TECD for domains under stress F1, F2, F3 and F4 has been displayed. Also TECD shows that recorded domains gradually dissolve in contiguous states with time gradient, attributing that recorded information is readable with time. **Bottom:** Each TECD image has its corresponding topographical image displaying no topographical alteration with time.

**Figure 7:**
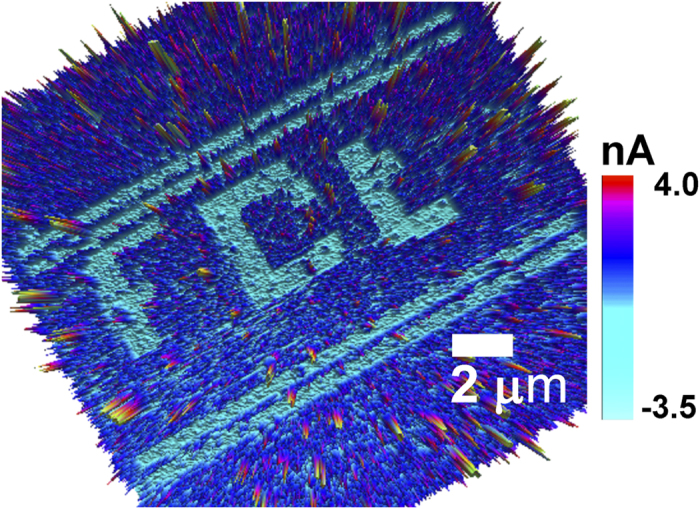
3D image of mechano-electrical writing on polyaniline surface based on CEM.

Now we have recorded different section on polymer surface by applying different stresses on it. Time evolutional current distribution (TECD) after recording in four different areas (1 ×1 μm^2^) with different stresses has been shown in Fig. 6. TECD has been visualized for t = 0 to 4 h, with interval of 1 h. Each TECD figure has its corresponding topographical image at the bottom. From the recorded scanning image it has been displayed that there is no topographical alteration with time changing form t = 0 to 4 h. This suggests that the polymer surface topography has not been distorted during the contact electrification mechanism and therefore can be inferred that the entire mechanism is related with the electronic dynamics within the polymer and polymer-metal interface barrier. In Fig. 6, the TECD image elucidates that each area is individualized by the current distribution disintegration between ploughed and non-ploughed areas. Regions of red (in colour) in TECD images indicate lesser current distribution, alluding different information which has been recorded through CEM with the AFM probe by applying different forces. Region *F*_*1*_ (=1.5 nN), *F*_*2*_(=3 nN) *F*_*3*_(=4.5 nN) and *F*_*4*_ (=6 nN) have therefore been ploughed by AFM probe in contact scanning mode with different forces. Time evolutional scanning shows that the *F*_*1*_, *F*_*2*_, *F*_*3*_ and *F*_*4*_ regions dissolve in contiguous states with time gradient, attributing that the recorded information is readable with time. Therefore, we have successfully open up a way to non-destructive nanoscale patterning of polyaniline surface using mechano-electrical energy harvesting by the *in-situ* AFM characterization in contact mode. By applying different contacting forces, the stress-induced information could be patterned on polymer surface, analogous with nanoscopic writable-readable data sheet. Moreover, Fig. S3 in supplementary information indicates that the recorded information on the polymer surface is also dependent on the number of repeated rubbing at the same area. Figure 7 shows a mechano-electrical image patterning on polymer surface. The study shows a promising application towards the self-powered writable-readable nanoscopic data sheet.

Additionally to investigate any effect of the ITO back electrode, we have performed CEM study of PANI films synthesized on silicon and gold coated silicon substrates. The same effect of contact electrification mechanism has been observed for all the substrates. In supplementary information, Fig. S6 shows the corresponding CEM study on silicon substrate.

## Discussion

In summary, we have demonstrated a stress induced non-destructive mechano-electrical polymer data sheet that is directly writable employing CEM instead of applying electrical bias. Recorded data on the polyaniline surface has been differentiated by the applied force during contact electrification. As the surface itself harvests energy, it can be claimed that the system behaves as a self-powered nanoscopic writable-readable device. TECD study shows the response of readability of the recorded data with time. These results not only underscore the importance of stress-induced patterning of polymer surface, but also the way out to scale up the self-powered device. All the measurements have been reported by exerting *in-situ* method to quantitatively characterize the contact electrification at nanoscale on polymer surface via a combination of AFM and conductive AFM in contact mode. We have systematically investigated the *in-situ* charge transfer through metal-polymer interface, the efficiency of current harvesting with respect to applied stress on the polymer by AFM probe, charge mobilization and scarcity from time evolutional multi-scanning. Eventually, this type of power generation by exploiting CEM can also be attractive as a new renewable source for alternative power application device.

## Methods

### Material synthesis

#### Reagents

Aniline (An) monomer (Aldrich) was distilled under vacuum prior to use. Ammonium persulfate (APS; 99.99%, Aldrich) and dopant D-10-camphorsulfonic acid (D-CSA, Aldrich) were used as received without further purification. Deionized (DI) water was used throughout.

#### Preparation of thin PANI films on different substrates

The thin films of PANI were deposited on different substrates like ITO-coated glass, *n*-type Si and gold coated silicon (the substrates were cleaned and processed following standard protocols) by a typical chemical oxidative polymerization of aniline at 0 ^o^C (ice bath) using APS as the oxidant in presence of D-CSA (dopant). Aniline and CSA were added to 20 mL DI water to make a colorless solution cooled at 0 ^o^C. The amount of CSA and An depended on their concentration and molar ratio (e.g., 1:2 or 1:1). The oxidant APS (used the same number of moles as the number of moles of An monomer) was dissolved in 10 mL DI water and precooled to add with the previous mixture with 10 s shaking. The polymerization was allowed to continue unagitated for 1 h (PANI-1) or more half an hour for PANI-2. A uniform dark green film was obtained after chemical deposition for PANI-1 (ES form) and or blue colour film for PANI-2 (more oxidized). The films were finally rinsed several times with DI water and methanol and then blow dried with compressed air before using in any further investigation. The thickness of the films was found approximately 235 nm, measured from the cross-sectional FIB image. It was calculated by AFM measurement that the r.m.s values of roughness of the films were approximately in the range of 12–30 nm (measured from the area of 4 × 4 μm^2^). To investigate the effect of surface roughness and others irregularities, we performed a series of experiments for over 20 different samples and also with different areas of them.

### Characterizations and measurements

Morphological studies were carried out with the images taken by a field emission scanning electron microscope (FESEM-FIB, Carl Zeiss Auriga Crossbeam microscope). The films were further characterized by Fourier transformed infrared (FTIR) spectroscopy and UV-vis-NIR spectroscopy (Shimadzu, UV-3101PC). FTIR spectra for the samples have been acquired using an attenuated total reflectance (ATR) sampling accessory (Smart iTR) equipped with a single-bounce diamond crystal on a Thermo Nicolet 6700 Spectrometer (used in the absorbance mode at 50 scans with a resolution of 4 cm^−1^ in the frequency range of 400–4000 cm^−1^).

#### AFM analyses

All AFM measurements were done in an Asylum Research MFP-3D Stand-alone using commercial *Pt-Ir* tip coated probes. All the data recorded and measurements done under different applied forces imply that the probe was throughout calibrated beforehand. The measured force is F = kδ × ∆v, The inverse optical lever sensitivity (δ) and spring constant (k) are both calibrated in one step, with accuracy. ∆v is the difference between free air probe deflection and contact deflection. All the measured data was analyzed offline with the Asylum Research tools developed within the IGOR Pro 6.22A data analysis software.

## Additional Information

**How to cite this article**: Goswami, S. *et al.* Stress Induced Mechano-electrical Writing-Reading of Polymer Film Powered by Contact Electrification Mechanism. *Sci. Rep.* **6**, 19514; doi: 10.1038/srep19514 (2016).

## Supplementary Material

Supplementary Information
