# Supplementary material for: Stress Induced Mechano-electrical Writing-Reading of Polymer Film Powered by Contact Electrification Mechanism
Source: Sci Rep. 2016 Jan 20;6:19514. doi: 10.1038/srep19514 (PMC4726212; doi:10.1038/srep19514)
Supplement: Supplementary Information [file srep19514-s1.pdf]

Supplementary Information

Stress Induced Mechano-electrical Writing-Reading of Polymer Film Powered by Contact Electrification Mechanism

Sumita Goswami, Suman Nandy*, Tomás R. Calmeiro, Rui Igreja, Rodrigo Martins and Elvira Fortunato*

i3N/CENIMAT, Department of Materials Science, Faculty of Science and Technology, Universidade NOVA de Lisboa and CEMOP/UNINOVA, Campus de Caparica, 2829-516 Caparica, Portugal

Corresponding authors E-Mail: snandy_ju@yahoo.co.in (SN), emf@fct.unl.pt (EF)

FTIR and XRD Study:

FTIR spectra of the samples are depicted in Fig. S1(a). As expected, some typical characteristics absorption bands of doped PANI were observed in the spectra. For instance, 1582-1585 and 1493-1496 cm^{-1} (due to stretching vibration of C═C in Quinoid (Q) and Benzenoid (B) rings respectively), 1305-1316 cm^{-1} (attributed to π-electron delocalization induced in the polymer by protonation of the doping process), 1248-1251 cm^{-1} (associated with the C─N stretching of the secondary aromatic amine), 1148-1155 cm^{-1} (due to the aromatic C─H in-plane bending), as well as, 828-832 cm^{-1} (related to the out-of-plane deformation of C─H in the 1, 4-disubstitued benzene ring)[1] were observed. The peaks observed at 802-805 and 1030-1038 cm^{-1} are attributable to the presence of sulphonic acid (─SO_3H) group indicating that as-prepared PANI-samples were doped with CSA.[2] Thus, the

FTIR spectra of PANI-1 and PANI-2 films display essentially the same absorptive characteristics as that of doped pristine PANI. But, the quinoid (Q)/benzenoid (B) ratio determined from the FTIR spectra (which is greater for PANI-2 than PANI-1) signify that more oxidized pernigraniline segments exist in sample PANI-2 whereas more doped emeraldine salt segments exist in case of PANI-1.[3] Also, the characteristics peaks corresponding to the aromatic rings for PANI-2 have been slightly shifted to the higher wave number side due to higher level of oxidation.

Both the forms of polyaniline films (PANI-1 and PANI-2) were amorphous in nature. There is no crystalline phase of polyaniline thin film as is shown in Fig. S1(b). The only peaks detected are from the polycrystalline structure of ITO film.

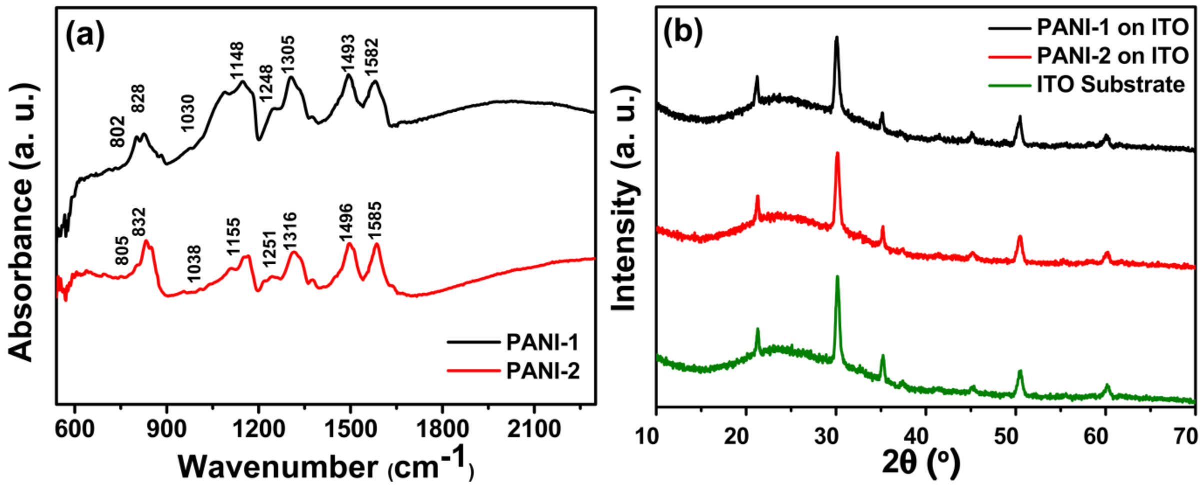

Figure S1: **(a)** FTIR analysis of PANI-1 and PANI-2 films. **(b)** XRD analysis of PANI-1 and PANI-2 films deposited on ITO substrate. For comparison purposes XRD of the ITO coated glass substrate is also presented.

Study on Contact Electrification Mechanism (CEM):

Fig. S2 exhibits a systematic investigation of electrical response for eight consecutive contacting forces with gradual increment amount without applying any electrical bias. Each force was holding up for 1 s at the time of encounter between AFM probe and polyaniline surface. It has been observed that the electrical response is increasing exponentially with the increment of applying force. The nature is quite similar with the nature of short circuit current (I_{SC}) for different applied bias. We did similar experiment for several different position of the PANI film to test the consistency in acquired data. Also the similar experiment was performed for different time scale.

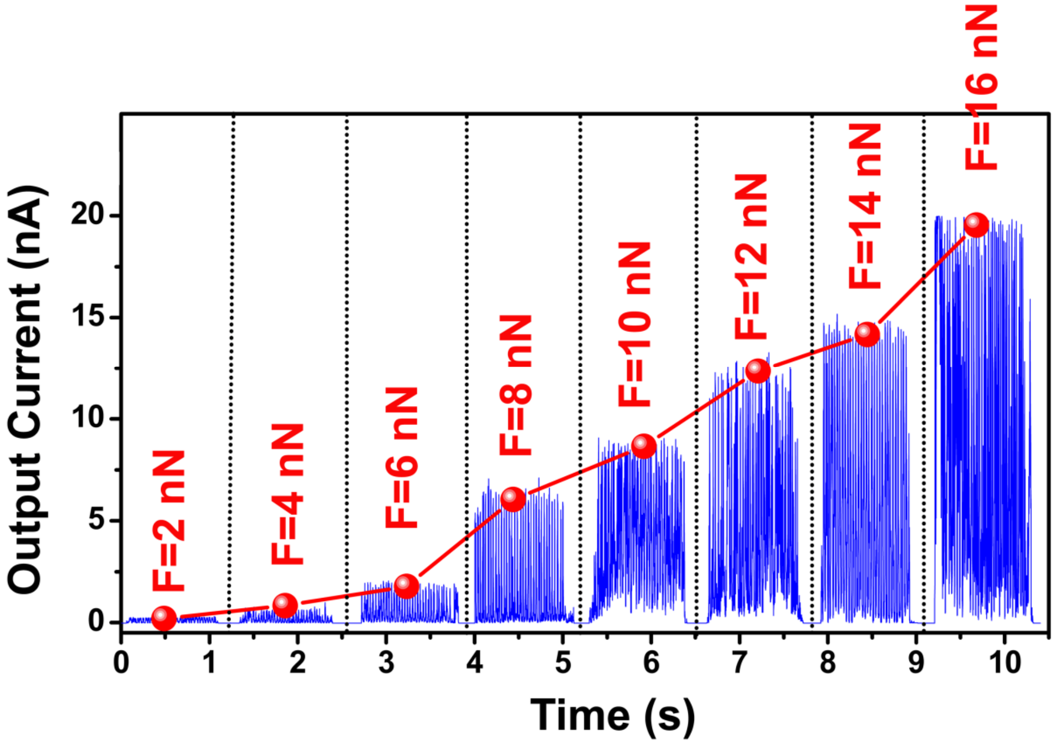

Figure S2: The measured electrical response for increasing amount of contacting force from 2 to 16 nN. Each applying force was conferring for 1s.

Regardless of the nature of stress induced charge transfer, Fig. S3 shows that the amount of charge transfer also depends on the number of cycle of rubbing. It is clear that the scarcity of charge carrier is increasing with increasing amount of repetition ploughing of the same area by AFM probe. This can be comprehended that the entire stress faced by the particular area of the polymer surface is increased by the number of cycle of rubbing. Also with each cycle of rubbing, a localized electrostatic field has been generated at the probe-surface contacting area due to CEM, which inhibits further charge transfer at the interface. This also makes a difference in recorded TECD though the topographical evolution with time remains unchanged.

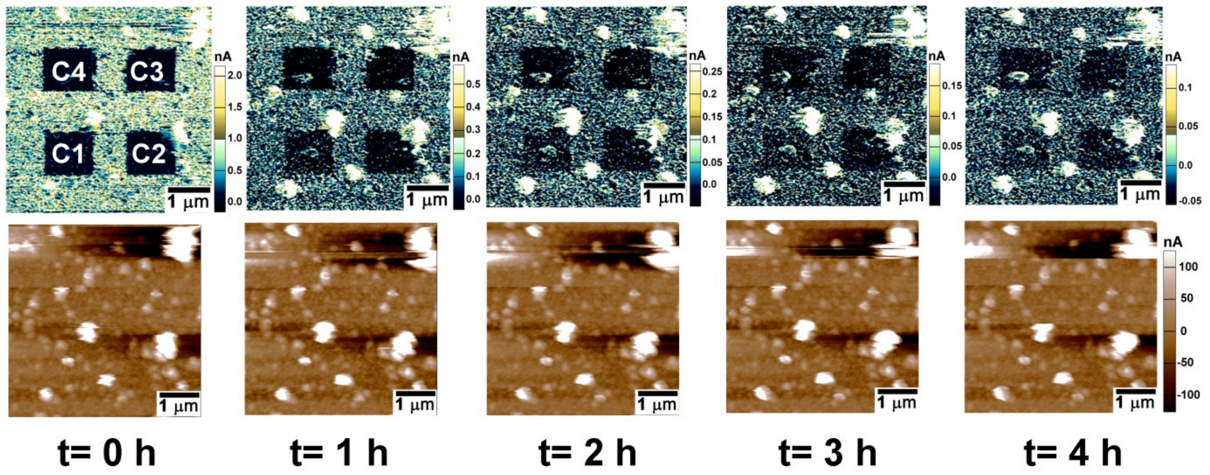

Figure S3: Top: Shows series of TECD of four separate domains (1×1 μm^2), ploughed under different cycles of rubbing. Domains of C1, C2, C3 and C4 are indicated by the number of repetitive rubbing. C1: Cycle of rubbing =1; C2: Cycle of rubbing =2; C3: Cycle of rubbing =3; C4: Cycle of rubbing =4. TECD has been recorded for time interval from t=0 to 4 h. **Bottom:** Each TECD image has its corresponding topographical image displaying no topographical alteration with time.

CEM patterning by imposing electrical bias:

We have also performed mehano-electrical patterning by imposing positive and negative electrical bias. A 2×2 μm^2 domain as shown in Fig. S4(a) and (b) has been ploughed by AFM probe in certain contacting force but with activation of +200 mV and -200 mV electrical bias respectively. Data recording was scanned in a 4×4 μm^2 area in contact mode without electrical bias, keeping the ploughed domain in the middle. Square in red of each image shows the counts of electrical response of the respective patterned domain. An equable counts of each mechano-electrical patterned domain based on CEM with ±200 mV electrical bias suggested that the mechanism is stress induced. There is no influence of electrical bias.

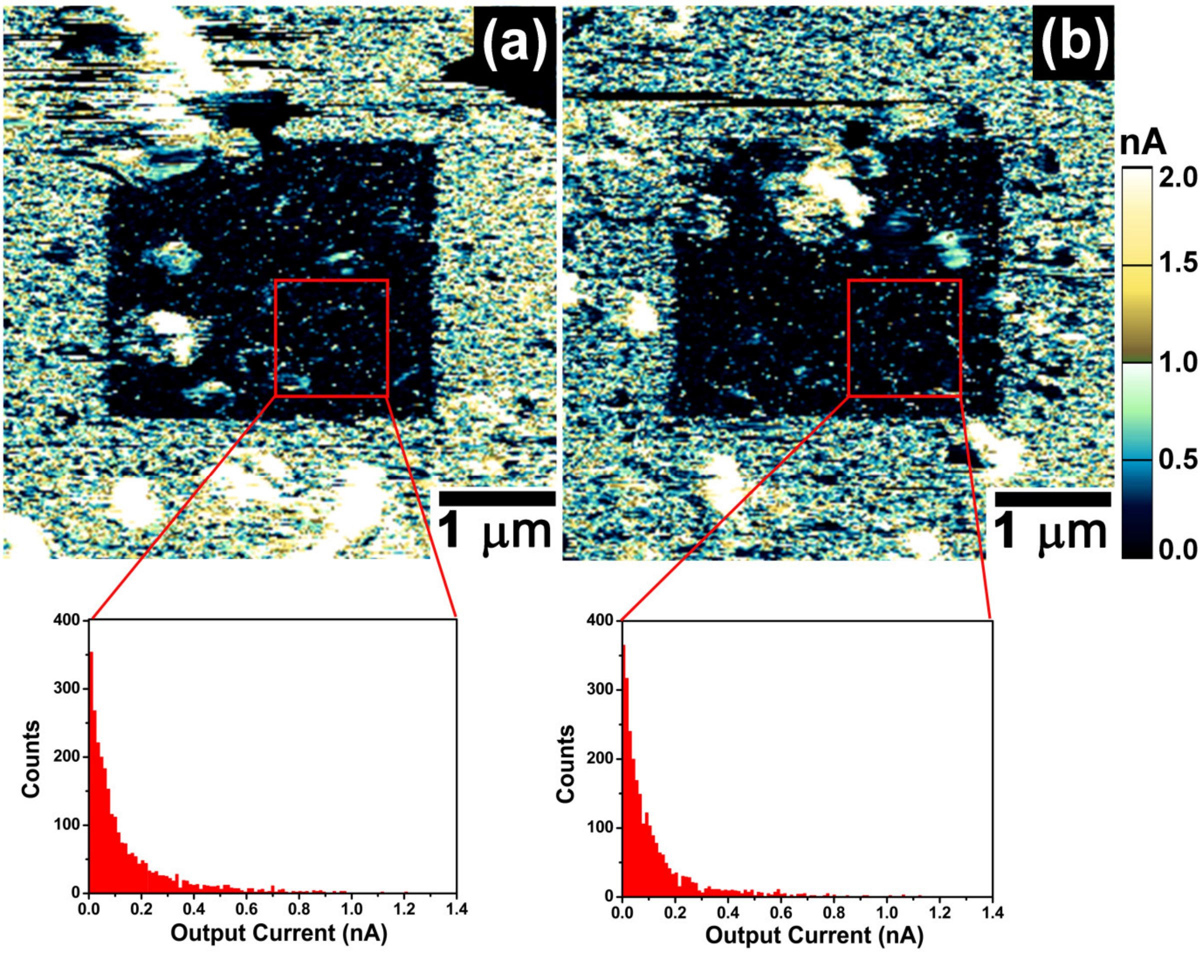

Figure S4: Exhibits a distribution of currents from the PANI-1 surface after ploughing centre domain (2×2 μm^2) by AFM probe in contact mode with electrical bias **(a)** +200 mV and **(b)** -200mV.

Study of surface potential after CEM pattering:

To investigate the effect of surface potential due to stressed induced contact electrification mechanism, PANI-1 film has been characterized by kelvin probe force microscopy (KPFM). Though the KPFM is a non-contact mode measurement where all over our measurement was in contact mode to observe the stress induced effect on the PANI surface. Therefore we first ploughed 2×2 μm^2 area in contact mode by AFM probe and then measured the surface potential of 5×5 μm^2 area by keeping the plough area in centre. Due to the locally built-up electric potential on the ploughed area arising from the contact electrification, we found a significant potential difference between ploughed and non-ploughed areas. Fig. S5 shows the uneven charge distribution over PANI-1 surface and the corresponding contact potential difference (CPD) profile.

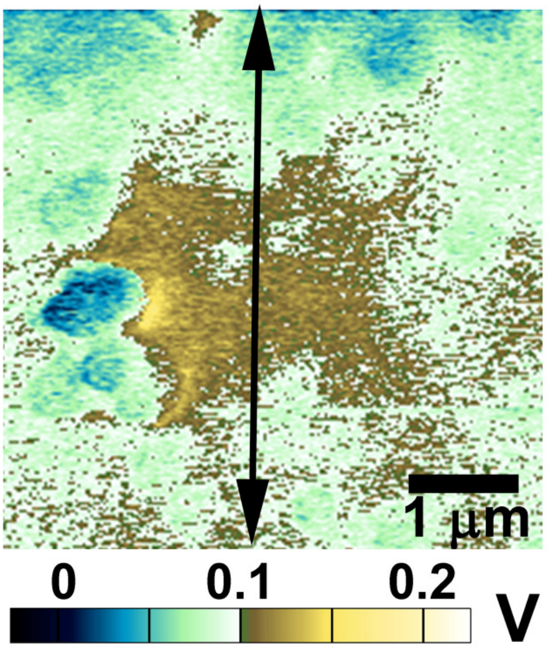

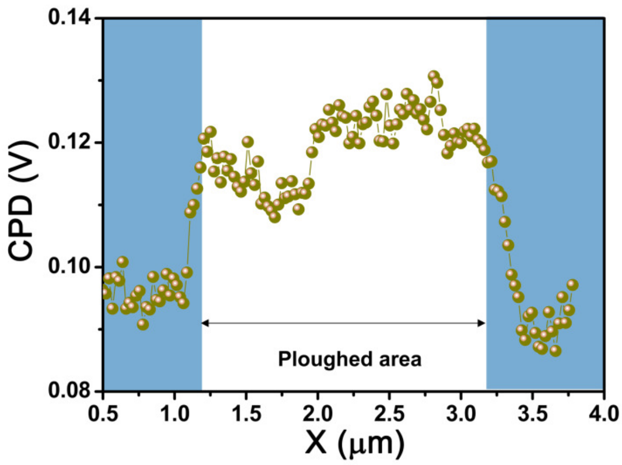

Figure S5: Left: Exhibits surface charge distribution over PANI-1 surface after ploughing the 2×2 μm^2 of centre area . **Right:** Shows the corresponding contact potential difference between ploughed (the regions indicated by white colour) and non-ploughed (the regions indicated by blue colour).

Similar as on ITO, we have also deposited PANI-1 on silicon substrate at the same time in chemical reaction. The results exhibit the same nature as the PANI-1 on ITO. The square domain (in dark red colour) in the centre of Fig. S6 (Top) is ploughed area, which has been rubbed with 7.5 nN contacting force. Exploring with the corresponding profile, it has been shown the current distribution at the ploughed domain is much lower than the neighbours. Yellow region in profile helps to differentiate the region. As we have already discussed that the ploughed domain has faced scarcity of free carries (electron) with generation of localized electrostatic field which then withhold further electrical transition.

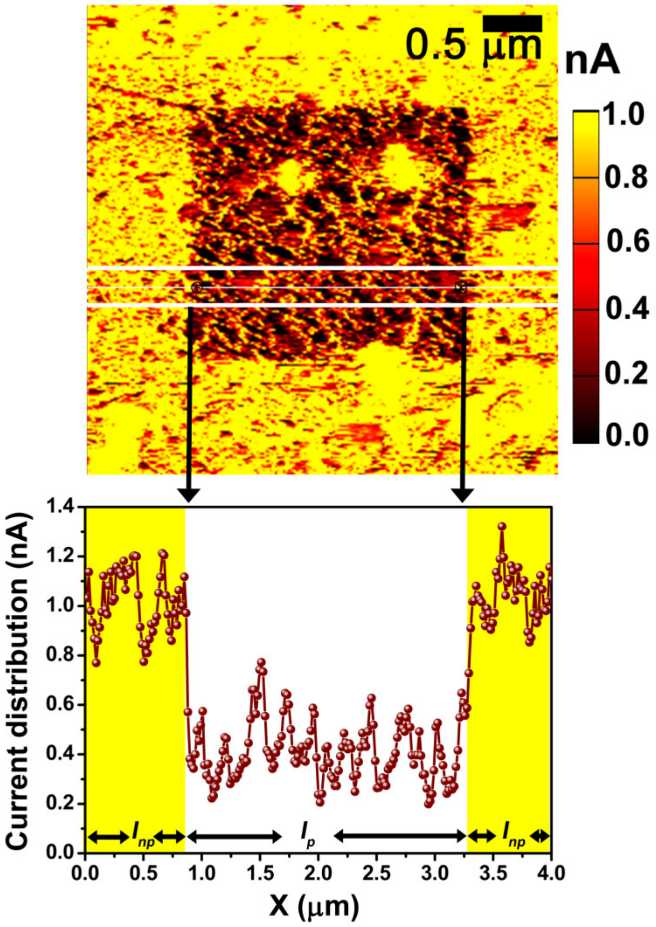

Figure S6: Exhibits an accumulation from PANI-1 surface, deposited on silicon (*n*-type doped). **Bottom:** Shows the corresponding current distribution profile (the regions indicated by the white line in top image).

References

1. Trchova, M.; Stejskal, J.; Polyaniline: The infrared spectroscopy of conducting polymer nanotubes. *Pure Appl. Chem.*, **83,** 1803-1817 (2011).

2. Saravanan, S.; Joseph Mathai, C.; Anantharaman, M.R.; Venkatachalam, S.; Prabhakaran, P.V.; Investigations on the electrical and structural properties of polyaniline doped with camphor sulphonic acid. *J. Phys. Chem. Solids*., 67, 1496-1501 (2006).

3. Quillard, S.; Louarn, G.; Lefrant, S.; Macdiarmid, A. G.; Vibrational analysis of polyaniline: A comparative study of leucoemeraldine, emeraldine, and pernigraniline bases. *Phys. Rev. B*, **50,** 12496-12508 (1994).
